# The influence of ecological and life history factors on ectothermic temperature–size responses: Analysis of three Lycaenidae butterflies (Lepidoptera)

**DOI:** 10.1002/ece3.5550

**Published:** 2019-08-14

**Authors:** Rebecca J. Wilson, Stephen J. Brooks, Phillip B. Fenberg

**Affiliations:** ^1^ Ocean and Earth Science, National Oceanography Centre Southampton University of Southampton Southampton UK; ^2^ Department of Life Sciences Natural History Museum London UK

**Keywords:** body size, ectotherm, Lepidoptera, museum collections, temperature, voltinism

## Abstract

Body size has been shown to decrease with increasing temperature in many species, prompting the suggestion that it is a universal ecological response. However, species with complex life cycles, such as holometabolous insects, may have correspondingly complicated temperature–size responses. Recent research suggests that life history and ecological traits may be important for determining the direction and strength of temperature–size responses. Yet, these factors are rarely included in analyses. Here, we aim to determine whether the size of the bivoltine butterfly, *Polyommatus bellargus*, and the univoltine butterflies, *Plebejus argus* and *Polyommatus coridon*, change in response to temperature and whether these responses differ between the sexes, and for *P. bellargus*, between generations. Forewing length was measured using digital specimens from the Natural History Museum, London (NHM), from one locality in the UK per species. The data were initially compared to annual and seasonal temperature values, without consideration of life history factors. Sex and generation of the individuals and mean monthly temperatures, which cover the growing period for each species, were then included in analyses. When compared to annual or seasonal temperatures only, size was not related to temperature for *P. bellargus* and *P. argus*, but there was a negative relationship between size and temperature for *P. coridon*. When sex, generation, and monthly temperatures were included, male adult size decreased as temperature increased in the early larval stages, and increased as temperature increased during the late larval stages. Results were similar but less consistent for females, while second generation *P. bellargus* showed no temperature–size response. In *P. coridon*, size decreased as temperature increased during the pupal stage. These results highlight the importance of including life history factors, sex, and monthly temperature data when studying temperature–size responses for species with complex life cycles.

## Graphical abstract

Life history and ecological factors are important for determining temperature–size responses of a species, but they are very rarely included in analyses. We show that sex and generation, as well as the use of appropriate temperature records, are important for understanding the temperature–size responses of three butterfly species. Within a species, temperature–size responses can vary between the sexes, generations, and immature stages.

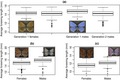

## 1 INTRODUCTION

Body size is considered to be one of the most important traits of an organism due to its strong links to ecology and life history (Baar, Friedman, Meiri, & Scharf, 2018; Bowden et al., 2015; Fenberg & Roy, 2008; Horne, Hirst, & Atkinson, 2015; McCauley, Hammond, Frances, & Mabry, 2015). Ecological rules such as the temperature–size rule (TSR) and Bergmann's rule (latitude‐size clines) have led to the prediction that body size declines will be a “universal” response to climate change (Daufresne, Lengfellner, & Sommer, 2009; Gardner, Peters, Kearney, Joseph, & Heinsohn, 2011). Specifically, the TSR predicts that individuals developing in cool conditions will be larger as adults than those developing in warm conditions, and this has been shown to be the case for many species (Daufresne et al., 2009; Gardner et al., 2011; Ghosh, Testa, & Shingleton, 2013; Horne et al., 2015; Irie, Morimoto, & Fischer, 2013; Ohlberger, 2013; Sheridan & Bickford, 2011; Tseng et al., 2018). However, it has also recently been shown that some species increase in size with increasing temperature, whereas some species appear to show no temperature–size response (Classen, Steffan‐Dewenter, Kindeketa, & Peters, 2017; Fenberg, Self, Stewart, Wilson, & Brooks, 2016; Horne et al., 2015; Høye, Hammel, Fuchs, & Toft, 2009; Scriber, Elliot, Maher, McGuire, & Niblack, 2014; Shelomi, 2012; Upton, Price, Percy, & Brooks, 2016). Thus, it is now clear that a reduction in body size is not a “universal” response to warming temperatures.

Recent studies have provided insights into the many ways ecological and life history factors are likely to affect the direction and strength of temperature–size responses (but a full synthesis is needed). These factors include, but are not limited to, sex, voltinism, trophic level, immature stage, and habitat type. For example, aquatic ectotherms exhibit a decrease in size with warming temperatures but the strength of response may vary depending on trophic level (Forster, Hirst, & Atkinson, 2012; Wilson‐Brodie, MacLean, & Fenberg, 2017). In contrast, some terrestrial species increase in size with warming temperatures, but response can vary based on sex and voltinism (Fenberg et al., 2016; Horne et al., 2015). In some holometabolous insects, univoltine species increase in size with temperature while multivoltine species decrease in size, whereas bivoltine species appear to show no change (Horne et al., 2015). While these are important findings, approaches thus far have not generally considered whether individual generations and/or sexes have varying responses. This can be significant because individuals from each generation will experience different temperatures. Fenberg et al. (2016) showed this is particularly important during growth of the final larval instar. Sex can also be an important factor especially if there is sexual size dimorphism (SSD), as males and females do not always respond in the same way to temperature (e.g., Fenberg et al., 2016; Høye et al., 2009). Thus, omission of such ecological and life history factors when conducting analyses may oversimplify the study system and/or cause some temperature–size responses to be masked.

Climate warming potentially provides a longer growing season for some insect species, particularly for temperate species emerging early in the season (Forrest, 2016). This is especially significant for obligate univoltine species (restricted to one generation per year) which often attain a larger size at maturation with increasing temperature (Horne et al., 2015). Some species, however, are able to increase voltinism and produce an extra generation per year at higher temperatures, resulting in a decrease in adult size due to a trade‐off between increased single generation growth and producing more generations with smaller individuals (Forrest, 2016; Horne et al., 2015; Van Dyck, Bonte, Puls, Gotthard, & Maes, 2015; Zeuss, Brunzel, & Brandl, 2016). Thus far, it is unclear how each generation of multivoltine species (more than two generations in one year) or obligate bivoltine species, which are restricted to two generations each year, will respond to increasing temperatures.

A recent study using museum collections and monthly temperature records over a 100‐year period of a British population of the univoltine butterfly species, *Hesperia comma*, found that adult size increased with increasing temperature; this, however, was only the case for males and not females, which showed no significant change in size (Fenberg et al., 2016). The females of this species are larger than males and therefore, these results suggest SSD decreases with increasing temperature. This differs from field research on the Arctic spider *Pardosa glacialis*, which found that females (but not males) increased in size with earlier snowmelt and the species became more sexually dimorphic in size (Høye et al., 2009), and from field research on two Arctic butterflies, *Boloria chariclea* and *Colias hecla*, which found that size was negatively correlated with temperature in both males and females despite the difference in size between the sexes (Bowden et al., 2015). These studies highlight both the importance of studying males and females separately, and that species may have different responses to climate change, reflecting different life history strategies, rather than following a particular rule.

Museum collections are a useful resource for studying historical size changes and can be used to create a time series which can be compared to climate variables over the same period (Lister et al., 2011). Furthermore, these collections often cover a wide range of taxa over large spatial areas and temporal periods (Johnson et al., 2011). There are, however, some potential problems in using museum collections as metadata are often incomplete or missing altogether, which reduces the number of useable specimens (Johnson et al., 2011; Lister et al., 2011), and specimens are usually collected opportunistically rather than systematically as part of long‐term projects (Kharouba, Lewthwaite, Guralnick, Kerr, & Vellend, 2018). Additionally, museum specimens often do not provide an ecological context, such as other species present or abiotic environmental factors, and therefore, the drivers of change may not be apparent (MacLean, Nielsen, Kingsolver, & Buckley, 2018). When species are well represented in collections, however, they provide a unique opportunity to study changes in species body size over a long time period (decades to centuries; Kharouba et al., 2018). As long as specimens are used critically (i.e., by disregarding those without sufficient metadata or that are in poor condition) and paired with appropriate temperature records, these collections can provide important insights into recent historical size changes, and may prove useful in predicting future changes.

Butterfly collections provide a useful resource for studying the effect of various factors on temperature–size responses. Good study species for this type of research are ones in which males and females, and in some cases separate generations, can easily be identified and climate data are readily available for analyses. This approach was used in previous research on *H. comma* (Fenberg et al., 2016) in which monthly temperature records were compared to adult size, rather than a single annual or seasonal temperature value. This has the advantage of assessing which monthly temperature has the most impact on adult size and therefore, in which life stage growth is most affected by temperature. Laboratory experiments often use a constant, single temperature per replicate (for example, studies used for meta‐analysis in Horne et al., 2015), but in the natural environment, temperature fluctuates and individuals may experience a wide range of temperatures during the growth period and from one year to the next as shown in field research by Bowden et al. (2015) and in historical data used by Fenberg et al. (2016).

In contrast to previous studies, which have not considered generational differences in temperature–size responses, we have chosen to examine how temperature affects the adult size of each generation in a bivoltine species, the Adonis Blue butterfly (*Polyommatus bellargus*), compared to the response of two univoltine species, one from the same habitat (the Chaklhill Blue butterfly *Polyommatus coridon*) and one from a different habitat type as *P. bellargus* (the Silver‐studded Blue butterfly *Plebejus argus*).* Polyommatus bellargus* has two temporally distinct generations (separated by approximately two weeks) and for all three species, the sexes are easily identifiable, and are present in large numbers in the collections of the Natural History Museum (NHM; London), making them ideal study species to investigate temperature–size responses over many decades.

Based on the meta‐analysis by Horne et al. (2015), we hypothesize that the univoltine species, *P. coridon* and *P. argus*, will increase in size with temperature and, as a bivoltine species, the size of *P. bellargus* will not change in response to temperature warming. Horne et al. (2015) did not include the effect of generation or sex in their analysis; however, life history traits, such as timing of the final larval stage and sex, can influence whether there is a response to temperature (Fenberg et al., 2016). Therefore, when sexes and generations are analyzed separately, we may find that a subset of the population is responding differently to temperature changes. If this is the case, we predict that for *P. bellargus*, generation one will show a greater size response to temperature than generation two due to the extended growing period available to generation one in warm years and the less favorable growing conditions during cool springs (Thomas, 1983; Thomas & Lewington, 2014). In particular, we would expect May temperatures to be important for generation one as this is when the species is in the final larval stage (Thomas & Lewington, 2014). For the two univoltine species, we would expect a similar response as *Hesperia comma* (Fenberg et al., 2016); therefore, we predict that the size *P. argus* and *P. coridon* will increase in size with increasing temperature for the month corresponding to the final larval stage (June for both species [Thomas & Lewington, 2014]), and there will be a greater size change for the smaller sex.

## 2 MATERIALS AND METHODS

### 2.1 Study system

The three study species are European Lepidoptera in the Lycaenidae with northern range edges in the UK. These species have different life history and ecological traits, which are summarized in Table 1. Further details about the species are given below.

**Table 1:** Some traits and characteristics of the three lycaenid study species (Brereton et al., 2008; Thomas, 1985, 1983; Thomas & Lewington, 2014)

Species	No. of generations	Larger sex	Habitat type	Overwinter stage	Relationship with ants	Larval food plant	Larval activity
*P. bellargus*	2	Males	Chalk hill grasslands	Larva	From second larval instar to pupal stage, March–October	*Hippocrepsis comosa*	Diurnal
*P. coridon*	1	Males	Chalk hill grasslands	Egg	As above, from May–August	*Hippocrepsis comosa*	Nocturnal
*P. argus*	1	Males	Heathland (also mosses, grassland and sand dunes)	Egg	All stages (egg to adult)	A variety of heath plants for example heathers or gorse	Nocturnal

In Britain, *P. bellargus* and *P. coridon* are restricted to calcareous grasslands on the south‐facing slopes of chalkhills, and populations are discrete (Brereton, Warren, Roy, & Stewart, 2008; Harper, Maclean, & Goulson, 2006; Thomas, 1983). *Plebejus argus* occurs on lowland heaths, mosses, calcareous grasslands, and sand dunes; this species has rapidly declined across the UK in the last century, and its stronghold is now on the New Forest heaths in Hampshire (Thomas, 1985; Thomas & Lewington, 2014). *Polyommatus bellargus* is obligatorily bivoltine, with the first generation emerging in May and June, and the second in August and September (Thomas, 1983). This makes *P. bellargus* an interesting study species as it will not respond to temperature by attempting to fit in an extra generation. In contrast to this, *P. coridon* and *P. argus* are both univoltine and adults are on the wing from mid‐July to early September and late June to August, respectively (Thomas, 1985; Thomas & Lewington, 2014). Despite laying their eggs on the same plant species, there is limited direct competition between larvae of *P. bellargus* and *P. coridon* as there is little overlap between the timings of the larval stages (Thomas, 1983). Like many European Lycaenidae species, *P. bellargus*, *P. coridon*, and *P. argus* are all associated with certain ant species, including *Lasius alumius*, *L. niger*, and *Myrmica sabuleti* (Fiedler, 1989; Kitching & Luke, 1985). The butterfly larvae produce secretions and are “milked” by ants. The ants nurture the larvae and bury them in earth cells when they are inactive or molting (Thomas, 1983; Thomas & Lewington, 2014).

### 2.2 Climate variables

Temperature records were obtained from the UK Meteorological Office (http://www.metoffice.gov.uk/climate/uk/summaries/datasets). Regional records of annual, seasonal (spring–summer average), and monthly mean temperatures were used from the South East and South‐central England (for *P. bellargus* and *P. argus*) and the East Anglia regions (for *P. coridon*), as these areas included our study sites. These data cover the years from 1910 to the present day and provided appropriate coverage for the majority of our butterfly data set.

### 2.3 Image analysis

The NHM has recently digitized their entire British butterfly and moth collections (Paterson et al., 2016). A large number of specimens of *P. bellargus* with accompanying collection date and locality metadata were present in the collections from Folkestone (51°4′53.05″N, 1°10′10.20″E; 1,396 out of 4,814 specimens for that species). The most common site in the collections for *P. coridon* was Therfield Heath (52°2′24.00″N, 0°1′12.00″W; 2,300 out of 3,097 specimens). For *P. argus*, many specimens were collected from the New Forest in Hampshire (centered around 50°52′12.00″N, 1°37′48.00″W; 1,049 out of 2,121 specimens). No other single area had as high a density of specimens for those species, and therefore, we focused on those locations to remove any potential effects of locality on the results.

Images of these specimens were checked for usability and separated into year groups and by generation. The generations of *P. bellargus* were separated following the methods used in Brooks et al. (2017). To be usable, the images needed to be sharp (i.e., not blurred) and in dorsal view with undamaged forewings. For *P. coridon*, some years had an excessive number of specimens so 50 usable specimens were selected at random for those years. The number of specimens used in analyses is given in Table 2, along with the years covered by the specimens. It should be noted that for *P. argus*, 48 specimens were not included as they were collected prior to 1910 (when local temperature records began). For *P. coridon*, males in 1919 were all considerably smaller than males in other years and therefore were excluded from analyses as outliers.

**Table 2:** The number of specimens used in data analyses for each species (total, males and females), the temporal range covered by those specimens, and the number of years included in the analysis within that range

Species	Total number	No. of males	No. of females	Date range	No. of years
*P. bellargus* (generation 1)	190	65	125	1912–1953	12
*P. bellargus* (generation 2)	532	203	329	1911–1956	18
*P. coridon*	417	133	284	1910–1931	15
*P. argus*	412	281	131	1910–1953	16

For each image, the sex of the specimen was noted and the length of the forewings were measured using ImageJ software. First, the scale was set in ImageJ for each individual image using the scale bar imbedded in each image to measure out a known distance, for example 10 mm. Each forewing was measured from the point at which the wing meets the thorax to the apex of the wing (not including the scales at the very edge of the wing as these were often absent), and an average forewing length was calculated for each individual. Forewing length of museum specimens has been found to correlate strongly with wing surface area (Fenberg et al., 2016) and has been used in previous studies as a proxy for overall body size (Bowden et al., 2015; Fenberg et al., 2016). Temperature data were added to the data sets for the corresponding years of collection.

### 2.4 Statistical analysis

Within each species (or generation where applicable), specimens were only included in the statistical analyses if there were three or more individuals for each sex per year. For each species, average forewing length was compared to mean annual temperature; all data were used in this model without taking into account generation or sex, which is consistent with methods used in previous studies and meta‐analyses of temperature–size responses (e.g., Baar et al., 2018; Horne et al., 2015). This was repeated using mean spring–summer temperatures, which were an average of March to August temperatures to include the main growing periods for all species.

Data were then analyzed using R statistical packages MASS and MuMMIn to run multiple linear regressions using two methods; stepwise regression in both directions to select variables for the final model, and information theoretic (IT) model selection with model averaging based on Akaike Information Criterion (AIC) as described in Fenberg et al. (2016). The linear models were used to determine whether there was a relationship between mean forewing length of individuals and monthly mean temperatures (for *P. bellargus*, each month from March to May for generation one and June to August for generation two; for *P. coridon* and *P. argus*, each month from March to July). These months were selected to cover the entire postwinter larval growth period and included the pupal phase as well (Thomas & Lewington, 2014). The data were analyzed separately for males and females, and for generations one and two in *P. bellargus*.

Additionally, where there were significant correlations between size and monthly temperatures, a percentage change in size per °C change in temperature was calculated for the most important months for predicting adult size so that change could be compared between species and sexes. Percentage wing size change was calculated using the formula (exp^(slope)^−1) × 100; slopes were calculated using the natual log of wing lengths to account for any scaling effects that may have resulted from the differences in size between species and sexes (Fenberg et al., 2016; Forster et al., 2012).

## 3 RESULTS

### 3.1 Sexual size dimorphism

**Figure 1:**
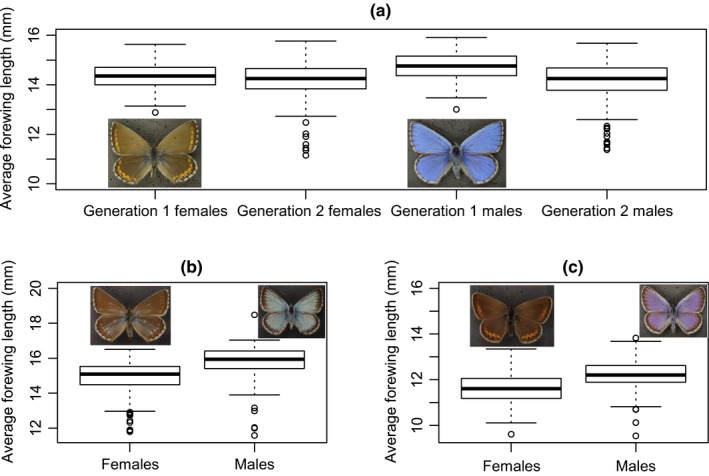
Boxplots of average forewing length of (a) *Polyommatus bellargus* specimens according to generation and sex, (b) *Polyommatus coridon* specimens according to sex, and (c) *Plebejus argus* specimens according to sex. The box represents the interquartile range, the line in the box represents the median value, the whiskers show the 5th and 95th quantiles, and the circles represent outliers. Images of each species are shown next to the relevant boxplots for both sexes

For *P. bellargus*, generation one adults were significantly larger than generation two adults (*F*_1, 718_ = 25.278, *p* < .001) but there was no overall difference in size between sexes (*F*_1, 718_ = 1.286, *p* = .257). The males of generation one were larger than females in generation one (*t* = −4.536, *p* < .001; male average size = 14.772 mm, female average size = 14.378 mm; Figure 1), but there was no significant difference in size between generation two males and females (*t* = −0.882, *p* = .378; male average size = 14.292 mm, female average size = 14.241 mm). Therefore, the SSD was limited to generation one, as a result of males being larger in this generation. For *P. coridon*, males were significantly larger than females (*t* = −3.7492, *p* < .001; male average size = 15.414 mm, female average size = 14.951 mm; Figure 1). This was also the case for *P. argus* (*t* = −7.995, *p* < .001; male average size = 12.231 mm, female average size = 11.683 mm; Figure 1).

### 3.2 Annual and seasonal temperature

Average forewing length was first compared to annual mean temperature using a linear model, irrespective of sex and generation, for each species. The model was not significant for *P. bellargus* (adjusted *R* ^2^ = .00224, *F* = 2.617, *df* = 1 and 720, *p* = .106) or *P. argus* (adjusted *R* ^2^ = .0045, *F* = 2.857, *df* = 1 and 410, *p* = .0917) but was significant for *P. coridon* (adjusted *R* ^2^ = .00904, *F* = 4.796, *df* = 1 and 415, *p* = .0291), which showed a negative relationship between size and annual temperature (slope estimate = −0.220). Forewing length was also compared with mean spring–summer temperature (an average of March to August temperatures), but was not significant for *P. bellargus* (adjusted *R* ^2 ^= −.000949, *F* = 0.316, *df* = 1 and 720, *p* = .574) or *P. argus* (adjusted *R* ^2^ = .00656, *F* = 3.716, *df* = 1 and 410, *p* = .0546). The model was, however, significant for *P. coridon* (adjusted *R* ^2^ = .0123, *F* = 6.159, *df* = 1 and 415, *p* = .0135), showing a negative relationship between spring–summer temperature and size (slope estimate = −0.244). However, these correlations for *P. coridon* may have been influenced by the significant correlations with July mean temperatures (*r* = .609, *p* = .0160; *r* = .677, *p* = .00560), which have a negative relationship with both male and female forewing length (see below), and to a lesser extent April temperatures, which had a negative effect on male forewing length (*r* = .629, *p* = .0120; *r* = .614, *p* = .0147).

### 3.3 Monthly mean temperature

#### 3.3.1 Polyommatus bellargus

**Figure 2:**
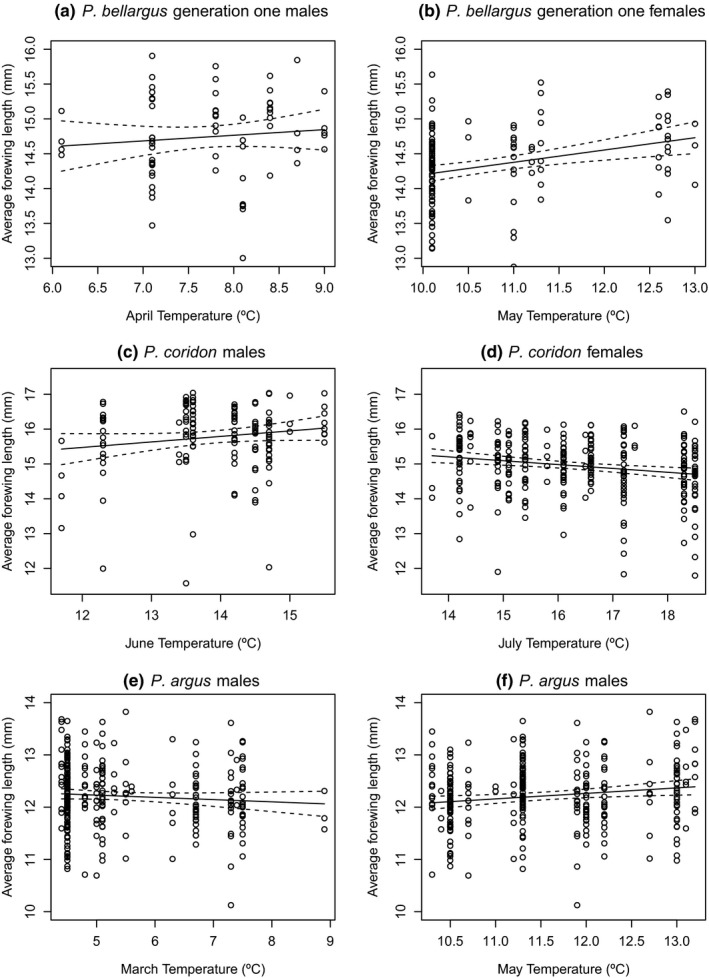
Average forewing length (mm) compared to mean monthly temperatures for (a) generation one *Polyommatus bellargus* males versus April temperature, (b) generation one *P. bellargus* females versus May temperature, (c) *Polyommatus coridon* males versus June temperature, (d) *P. coridon* females versus July temperature, (e) *Plebejus argus* males versus March temperature, and (f) *P. argus* males versus May temperature. Circles represent individual specimens, solid lines are the predicted values given by the linear models, and the dashed lines represent two standard errors above and below the predicted values. The months shown in the plots are those which were the most important for predicting adult size in the multiple linear regression analysis

**Figure 3:**
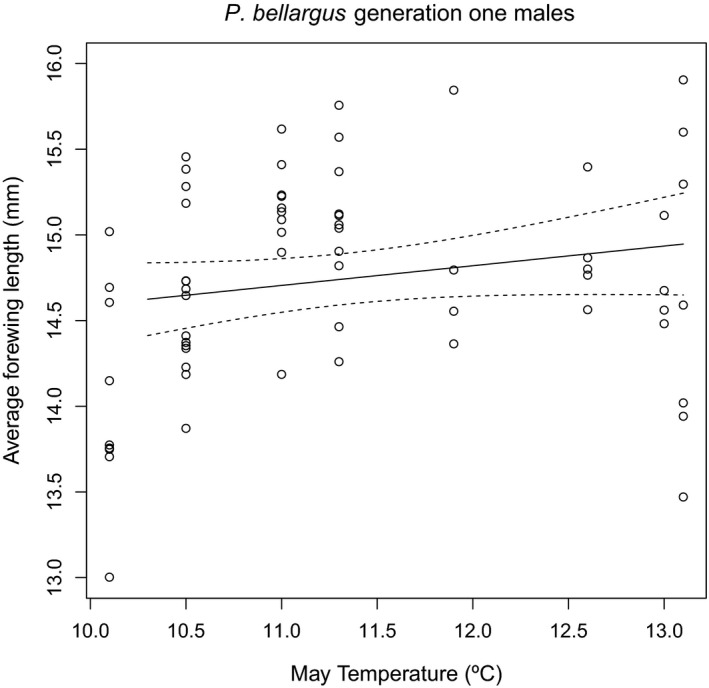
Plot of average forewing length (mm) versus May temperature for generation one *Polyommatus bellargus* males. Circles represent individual specimens, the solid line is the predicted values given by the linear model, and the dashed lines represent two standard errors above and below the predicted values. Note, the increasing trend during years covering cool to moderate May temperatures (10–11.5°C)

Multiple linear regression of the *P. bellargus* size data using mean monthly temperatures for males and females in both generations showed that the model was significant for generation one males (Table 3), with mean March, April, and May temperatures as significant variables; these variables were included in the final model after stepwise regression (Table 4a). For generation one females, the model was also significant (Table 3); May was the only significant month but March was also included in the final model after stepwise regression (Table 4a). In both cases, May temperature (and, for males, April temperature) had a positive relationship with forewing length (Table 4a; Figures 2a,b and 3), but March temperature had a negative relationship with forewing length as shown by the slope estimates (Table 4a). These results imply that adult size decreases as temperature increases during early larval stages, but adult size increases as temperature increases during late larval stages (Table 5). With increasing May temperatures, there was a 1.09% increase in adult size per °C for generation one males and a 0.97% increase for generation one females. For males, this positive relationship appears strongest during years when May temperatures are cool to moderate (~10–11.5°C; Figure 3). In addition, for males, there was also a 3.03% increase in size with April temperatures and a 2.02% decrease with March temperatures. The models were not significant for generation two males or females (Table 3).

**Table 3:** Results of the linear models for predicting average forewing length of *Polyommatus bellargus* (both generations),* Polyommatus cordion*, and *Plebejus argus* using mean monthly temperatures as variables; values are adjusted *R* ^2^ (AR^2^), the *F* statistic (*F*), degrees of freedom (*df*), and the *p*‐value (*p*)

	Males				Females			
	AR^2^	*F*	*df*	*p*	AR^2^	*F*	*df*	*p*
*P. bellargus* generation 1	0.147	4.67	3, 61	.00529	0.101	5.62	3, 121	.00122
*P. bellargus* generation 2	0.00534	1.36	3, 199	.256	−0.00820	0.111	3, 325	.954
*P. coridon*	0.0624	2.76	5, 127	.0213	0.0431	3.55	5, 278	.00397
*P. argus*	0.0408	3.38	5, 275	.00555	0.0466	2.27	5, 125	.0514

Note

Results are for models analyzing males and females separately.

**Table 4:** (a) Outputs for variables included in linear models for *Polyommatus bellargus* generation one males and females using mean monthly temperatures. (b) Outputs for variables included in linear models for *Polyommatus coridon* males and females using mean monthly temperatures. (c) Outputs for variables included in linear models for *Plebejus argus* males using mean monthly temperatures

	*t*‐value	*p*	Slope estimate	Standard error	Lower CI	Upper CI	Importance	VIF	Final model (Y/N)
(a)									
Males									
**March**	−3.178	.00254	−0.291	0.0956	−0.482	−0.100	0.94	2.286	Y
**April**	3.105	.00288	0.425	0.143	0.139	0.711	0.94	2.331	Y
**May**	2.275	.0264	0.157	0.0703	0.016	0.297	0.80	1.043	Y
Females									
**March**	−1.663	.0989	−0.117	0.0754	−0.266	0.0325	0.55	1.536	Y
April	0.720	.473	0.0394	0.0956	−0.150	0.228	0.29	1.285	N
**May**	2.660	.00886	0.159	0.0523	0.0550	0.262	0.97	1.232	Y
(b)									
Males									
**March**	1.627	.106	0.202	0.110	−0.0160	0.419	0.66	1.814	Y
April	−1.396	.165	−0.102	0.0995	−0.299	0.0942	0.39	1.600	Y
**May**	−1.806	.0733	−0.217	0.162	−0.536	0.103	0.52	3.459	Y
**June**	2.495	.0139	0.240	0.116	0.0110	0.469	0.77	1.573	Y
**July**	−1.870	.0638	−0.164	0.0972	−0.356	0.0278	0.62	1.871	Y
Females									
March	0.761	.447	0.0311	0.0453	−0.0581	0.120	0.31	1.650	N
April	−0.662	.509	−0.0229	0.0617	−0.144	0.0985	0.28	1.637	N
**May**	−2.232	.0264	−0.131	0.0564	−0.242	−0.0200	0.86	1.729	Y
June	0.048	.962	0.00555	0.0580	−0.108	0.120	0.26	1.330	N
**July**	−3.932	<.001	−0.150	0.0405	−0.230	−0.0706	1.00	1.533	Y
(c)									
Males									
**March**	−2.664	.00817	−0.0898	0.0403	−0.169	−0.0105	0.86	1.518	Y
**April**	1.397	.163	0.0634	0.0500	−0.350	0.167	0.46	1.476	N
**May**	2.336	.0202	0.124	0.0505	0.0251	0.224	0.89	1.606	Y
**June**	1.412	.159	0.142	0.0886	−0.0319	0.317	0.58	1.312	Y
July	−0.361	.718	−0.00468	0.0369	−0.0755	0.0678	0.27	1.253	N

Note

*t*‐value and significance (*p*) for each variable, slope estimate, standard error and upper and lower confidence intervals (CIs), importance scores, variance inflation factors (VIFs) and whether the variable was selected for the final model after stepwise regression in both directions. Slope estimates, standard error, and confidence intervals are based on an average of all candidate models (using the IT‐AIC approach), and variables in bold are those retained after nested models were removed. Importance scores are based on the number of candidate models the variable was present in, with a score of 1.0 indicating that the variable was present in all candidate models.

**Table 5:** Stage‐specific temperature–size trends in three lycaenid spcies, and *Hesperia comma* (Hesperiidae; results from Fenberg et al., 2016)

Life stage	Effect of increasing temperature on size	*P. bellargus* (G1)		*P. coridon*		*P. argus*		*H. comma*	
		M	F	M	F	M	F	M	F
Early instar larvae	Decrease in size	Y	Y	Y	Y	Y	N	Y	N
Late instar larvae	Increase in size	Y	Y	Y	N	Y	N	Y	N
Pupae	Decrease in size	?	?	Y	Y	N	N	N	N

Note

Yes (Y) and no (N) indicate whether males (M) and females (F) of each species follow the stage‐specific trends (second column) appearing in the linear models (Table 4a–c) for the effect of temperature on adult size. The influence of temperature on adult size during the pupal stage in *Polyommatus bellargus* could not be assessed due to an overlap in timing with the final larval instar.

#### 3.3.2 Polyommatus coridon

For *P. coridon*, multiple linear regression models with mean monthly temperatures were significant for both males and females. For males, mean June temperature was the only significant variable, but all variables were included in the final model after stepwise regression (Table 4b). For females, mean July and May temperatures were significant variables and the only variables included in the final model after stepwise regression (Table 4b). The results imply that adult size in both males and females decreases in size as temperature increases during early larval stages, and adult males increase in size as temperature increases during late larval stages (Table 5). Additionally, the results suggest that both males and females decrease in size when temperatures are higher during the pupal stage. For males, there was a 1.93% increase in adult size per °C increase in June temperatures (Figure 2c) compared to a 1.10% decrease in adult size of females per °C increase in July temperatures (Figure 2d).

#### 3.3.3 Plebejus argus

The multiple linear regression was significant for male, but not female *P. argus* (Table 3). For the males, mean March and May temperatures were significant and were included in the final model after stepwise regression along with June temperatures (Table 4c). March temperatures had a negative relationship with forewing length (Table 4c; Figure 2e), but May and June temperatures had a positive relationship with forewing length (Table 4c; Figure 2f). This trend is the same as in the other two species resulting in a decrease in adult male size as temperature increases during early larval stages and an increase in size as temperature increases during late larval stages (Table 5). There was a 0.85% decrease in male adult size per °C increase in March temperatures compared to a 0.95% increase in male adult size per °C increase in May temperatures. Female *P. argus*, however, do not show this trend (Table 5).

## 4 DISCUSSION

Many insect species have seasonally complex life cycles (Kingsolver et al., 2011). As a result, individuals at different stages within each cycle can respond to seasonal temperatures in different ways; either by varying growth rates (during the larval phase), emergence time (during the pupal stage), and/or dispersal distances (during the adult stage; Fenberg et al., 2016). Moreover, responses to temperature can vary by sex, and, for those species with more than one generation per year, by generation. Although rarely studied, untangling how and why species with complex life cycles respond to seasonal temperature during each life cycle stage requires an approach that takes into consideration multiple ecological and life history factors. Here, we have taken such an approach by exploring one facet of temperature responses (body size changes) in three species with complex life cycles.

When data were analyzed with no consideration of sex or generation and only using one temperature measure for each year (annual or spring–summer averages), there was no significant relationship between size and temperature for the bivoltine species, *P. bellargus*. This is consistent with results shown in Horne et al. (2015). In contrast to those results, however, the univoltine species studied here did not increase in size when compared to a single temperature value; *P. argus* showed no response to temperature and *P. coridon* decreased in size with increasing temperature. Often these annual and seasonal values may be the only climate data available but they are biologically less meaningful than using the growth period monthly averages for short‐lived species (such as those studied here). As we show, the temperature–size relationship of an organism can also vary between and within life stages, which would not be detected unless temperature records for the appropriate months are included in analyses. This is important, since any significant effect obtained with annual or seasonal temperatures could result from a correlation with temperatures during one or more months during the life cycle, in which case the full complexity of the response to temperature will be missed. Furthermore, for species with more than one generation in a year, each generation may experience different environmental conditions (e.g., climate and food quality and quantity), which can differentially affect size between generations (Horne, Hirst, & Atkinson, 2017). If generational differences are not taken into account (e.g., by averaging wing length of all individuals regardless of generation), or if there is a bias toward a particular generation in the data set, especially if that generation is not responsive to temperature, any effects of temperature on size may be masked.

When growth period monthly temperatures, sex, and generation are included separately in the models for the three species, there is a significant relationship between size and temperature, apart from female *P. argus* and generation two *P. bellargus*, which showed no response to temperature. While there are varying responses within a species between males and females, generations and larval stages, temperatures during the late larval stages were generally found to be the most important for predicting adult size. This is consistent with previous results for other butterfly species (Fenberg et al., 2016; MacLean, Kingsolver, & Buckley, 2016).

Although temperatures during late and final larval stages are most predictive of adult size, we also find a general trend of decreasing adult size with increasing temperature during early larval stages (in line with the TSR). Previous research on the univoltine butterfly species *Hesperia comma* also found changes in male size with temperature which followed these trends (Table 5; Fenberg et al., 2016). For *P. coridon*, there is also a decrease in adult size when temperatures increase during the pupal stage. It was not possible to test the influence of temperature on the pupal stage in *P. bellargus* as some specimens may be in the pupal stage during May and others may be in the late larval stage. To a lesser extent, this may also be the case in *P. coridon* and *P. argus*. A decrease in size with increasing temperature during early larval stages may be due to a trade‐off between using energy for growth and energy for producing secretions to attract ants, which are more active during warmer conditions (Thomas & Lewington, 2014). Another possible explanation is that ecdysis (moulting) takes place more slowly and at a higher metabolic cost at lower temperatures (which is more likely earlier in the season), and therefore, early stage larvae in cool conditions take in more food over a longer period at a higher efficiency, and are able to grow to a larger size during early development (Karl & Fischer, 2008).

The increasing size in males following increasing temperature (i.e., showing reverse TSR) during late larval stages of each species studied here (and *H. comma*; Fenberg et al., 2016) may be the result of reproductive pressure. Males that emerge earlier have a competitive advantage for access to females. At cooler temperatures, there is a trade‐off between reaching a large size and emerging earlier. Therefore, males tend to be smaller during years when cooler conditions coincide with the late larval stages (Hirst, Horne, & Atkinson, 2015). Males must also actively compete with other males for females (in *P. bellargus* males swarm around freshly emerged females; Thomas & Lewington, 2014), so large males are at an advantage. However, emerging in time to compete for females may be more important for males than growing to a larger size, hence, they tend to be smaller in years with lower temperatures during late larval stages (e.g., Figure 3). For females, only *P. bellargus* generation one showed an increase in size following an increase in temperature during late larval stages. In bivoltine (and multivoltine) species, females in the first generation may be under time pressure to emerge and lay eggs. Therefore, in cooler years, when growth is slower, females emerge at a smaller size.

Conversely, in univoltine species, females are under less time pressure to develop so may prioritize growth, to increase fecundity, and therefore female size may be less affected by temperature. Nevertheless, for female *P. coridon*, our results showed a decrease in size with increasing May temperatures, although it was smaller than the decrease in size shown by the males. May is towards the end of the period occupied by early larval stages in this species. A possible explanation is that the relationship with ants starts in May and, therefore, the larvae use some energy for producing the secretions that are attractive to ants, and this activity increases under warmer conditions (Thomas & Lewington, 2014). This may also be the reason for the decrease in adult size when higher temperatures occur during the pupal stage, because the pupa of this species also produces secretions and sounds to attract ants (Thomas & Lewington, 2014). This contrasts with results in a study of the univoltine butterfly *Anthocharis cardamines*, which is not associated with ants in the larval or pupal stages, which found that an increase in adult body size was correlated with temperature increases during the pupal stage of development, and that both sexes responded in the same way (Davies, 2019).

Our findings suggest that a reversal of the TSR occurs only during late larval instars for males in univoltine species and in both sexes in the first generation of bivoltine species. It is likely that the second generation of bivoltine species (and any further generations in multivoltine species) do not respond to temperature by changing size due to the limited time available for growth and conditions being more favorable for growth later in the season. For example, in second generation *P. bellargus*, the larval foodplant covers a larger area, the plants are taller, and the larvae are not restricted to the warmest short vetches (Thomas & Lewington, 2014). This is reflected in the greater abundance of individuals in generation two in the museum collections and in population monitoring data (Thomas, 1983; Thomas & Lewington, 2014; UKBMS, 2018). A phenological study of British butterfly species found that in warmer years, both generations one and two of *P. bellargus* emerge earlier than in cooler years (Brooks et al., 2017). Therefore, it is possible that any increase in growth rate due to increased temperature is counteracted by an earlier emergence time leading to no overall change in average size between years for generation two.

In contrast to our results, a meta‐analysis of a large number of arthropod species found that, in general, there was no difference in the strength and direction of the temperature–size responses in males and females of the same species, and therefore, SSD did not change with temperature (Hirst et al., 2015). Yet in agreement with other previous studies, our results show that temperature can have variable effects on the sizes of each sex, resulting in a shift away or towards SSD (Fenberg et al., 2016; Høye et al., 2009). Unlike most butterfly species, in which females are the larger sex and have a longer development time (Teder, 2014; Wiklund & Kaitala, 1995), the males in all three of our study species are larger, despite males emerging earlier than females (Thomas, 1985). Therefore, an increase in female size (as the smaller sex) with increasing temperature was predicted. Yet, for *P. argus* and *P. coridon*, males increased in size with increasing temperatures during the late larval stages, while the females did not (Table 5). A similar trend was found in *H. comma* (Table 5; Fenberg et al., 2016), despite males being smaller in that species, which suggests that sex‐specific life history constraints are more important than the direction of SSD for predicting which of the sexes will respond to temperature. Furthermore, for generation one *P. bellargus*, the percentage increases in male size with increasing April and May temperatures are larger than the increase in female size with increasing May temperatures. On the other hand, in *P. argus* and *H. comma*, males decreased in size as temperature increased during the early larval stages, whereas females did not (Table 5; Fenberg et al., 2016). Clearly, the sizes of both sexes can respond differently to temperature, which will have variable effects on SSD. This will partly depend on which months during the life cycle have temperatures that are higher or lower than average, and on the initial strength and direction of the SSD. As a future response to climate warming (when all months during larval stages are predicted to be warmer), if the growth rate during late larval stages is greater than the growth rate during early larval stages, SSD will become increasingly biased towards males, at least among four British butterfly species (including *H. comma*; Fenberg et al., 2016).

As our data were derived from museum specimens, not all ecological and life history traits could be considered in our analyses. An earlier study of the relationship between size and traits of multiple butterfly species found low (but significant) correlations between adult body size and several life history traits, including the proportion of the year adults are present, larval development time, and type of foodplant (Garcia‐Barros, 2000). *Plebejus argus*, for example, has some flexibility in food and habitat preference, which may be why the temperature–size responses in this species are not as strong as those of *P. bellargus* and *P. coriodon*. If quality or quantity of food plant species were affected by changes in temperature or other environmental conditions, this would be less problematic for *P. argus*, which could switch plants and therefore show a weaker size response to temperature. We were also unable to investigate how the relationship between butterfly larvae and the ant species may have been affected by temperature, which may have led to indirect impacts on adult butterfly size. *Plebejus argus* has the closest relationship with ants of the three species, and density of *P. argus* populations has been shown to positively correlate with ant nest density (Ravenscroft, 1990). An additional factor to consider is the life cycle of the species. *Polyommatus bellargus*, for example, overwinters as a larvae whereas *P. argus* and *P. coridon* overwinter as eggs (Thomas & Lewington, 2014). Yet, they are all direct developers (i.e., there is no pupal diapause) and therefore may be subject to more seasonal time constraints and variation than species which diapause in the pupal phase, such as *A. cardamines* (Davies, 2019). This may explain why all three lycaenid species we have studied, and *H. comma*, respond to temperature in the larval stage (Table 5; Fenberg et al., 2016) but the pierid butterfly *A. cardamines*, which overwinters in the pupal stage, instead responds to temperature in the pupal stage (Davies, 2019).

Finally, we note that, while climate change is resulting in warmer mean day‐time air temperatures and the earlier emergence of many butterfly species, our study does not take into account the effect of night‐time temperatures or temperatures at ground level on size. Advances in phenology may result in certain life stages (particularly early larval stages) being exposed to shorter days, so that, although day‐time temperature is higher, these stages will be exposed to cooler night‐time temperatures for longer. This is particularly relevant for *P. coridon* and *P. argus*, which are nocturnally active (Thomas & Lewington, 2014). Yet, it is likely that ground temperatures, especially around the larval host plants, will increase even during the night if day‐time temperatures are higher, which is probably why these two species show the same general temperature–size trends as *P. bellargus*, which has diurnal larvae.

## 5 CONCLUSIONS

Temperature–size responses can vary according to life cycle factors (e.g., voltinism and life history stage) and sex. If these factors are not included in analyses, adult size of a species may not appear responsive, especially when compared to annual or seasonal temperatures. However, a temperature–size response can emerge if studies separately analyze sex, generation, and life history stage with monthly temperatures during growth. While the species studied here all responded in some way to temperatures during immature stage development, the responses varied in strength and direction. Generation one male and female *P. bellargus* and males of *P. coridon* and *P. argus* responded in a similar way to previously studied univoltine species (Fenberg et al., 2016; Horne et al., 2015) and became larger in years with warmer temperatures during late larval stages and, therefore, showed the reverse of the TSR. Conversely, adult body size decreased in size with increasing temperature during early larval instar development and, in *P. coridon*, during pupal development, in line with the TSR. This suggests that, within a species, the temperature–size response can change direction as the immature stages progress (Table 5). Furthermore, these results suggest that for species which follow this trend, there will likely be an increase in SSD due to climate warming, if increases in size during late larval instars are greater than decreases in size during early larval instars. This not only highlights the importance of integrating life history factors into temperature–size response analyses, but also emphasizes that size declines in response to climate warming are not “universal” and that the TSR is too simplistic, especially for species with complex life cycles.

While not all aspects of ecology can be included directly in a study using museum specimens, these collections can provide useful insights into the responses of organisms to temperature change in the recent historical past. These data, when used in combination with monthly temperature records, can unravel the complex interactions between individuals and some of the factors that control body size. Furthermore, museum specimens provided the best opportunity to study the size response of these species to a wide range of temperatures as field studies would require continuous monitoring over many years and laboratory experiments would be hard to conduct due to the strong relationship between the juvenile stages of these species and ants.

Future studies examining temperature–size responses for species with complex life histories should be aware that the strength and direction of temperature effects may vary according to growth stage, sex, and generation. Where possible, studies using natural history collections can be used in combination with field studies and laboratory experiments to give a more holistic approach to studying temperature–size responses and predicting how these responses may be affected by climate change.

## CONFLICT OF INTERESTS

There are no competing interests to declare.

## AUTHORS' CONTRIBUTIONS

All authors contributed in conceiving the ideas, designing methodology, and writing, and agreed to the final manuscript; RJW collected and analyzed the data, and led the writing of the manuscript.

## Data Availability

Data available from the Dryad Digital Repository: https://doi.org/10.5061/dryad.f5cf448.
